# Control of Methicillin-Resistant *Staphylococcus aureus* Strains Associated With a Hospital Outbreak Involving Contamination From Anesthesia Equipment Using UV-C

**DOI:** 10.3389/fmicb.2020.600093

**Published:** 2020-12-14

**Authors:** Sara A. Ochoa, Ariadnna Cruz-Córdova, Jetsi Mancilla-Rojano, Gerardo Escalona-Venegas, Veronica Esteban-Kenel, Isabel Franco-Hernández, Israel Parra-Ortega, José Arellano-Galindo, Rigoberto Hernández-Castro, Citlalli F. Perez-López, Daniela De la Rosa-Zamboni, Juan Xicohtencatl-Cortes

**Affiliations:** ^1^Laboratorio de Investigación en Bacteriología Intestinal, Hospital Infantil de México Federico Gómez, Ciudad de México, Mexico; ^2^Facultad de Medicina, Posgrado de Ciencias Biológicas, Universidad Nacional Autónoma de México, Ciudad de México, Mexico; ^3^Laboratorio Central de Bacteriología, Hospital Infantil de México Federico Gómez, Ciudad de México, Mexico; ^4^Departamento de Infectología, Hospital Infantil de México Federico Gómez, Ciudad de México, Mexico; ^5^Departamento de Ecología de Agentes Patógenos, Hospital General Dr. Manuel Gea González, Ciudad de México, Mexico; ^6^Departamento de Epidemiología Hospitalaria, Hospital Infantil de México Federico Gómez, Ciudad de México, Mexico

**Keywords:** MRSA, multidrug resistance, PFGE, MLST, genetic diversity

## Abstract

Methicillin-resistant *Staphylococcus aureus* (MRSA) is considered an opportunistic pathogen in humans and is mainly associated with healthcare-associated infections (HCAIs). This bacterium colonizes the skin and mucous membranes of healthy people and causes frequent hospital outbreaks. The aim of this study was to perform molecular typing of the staphylococcal cassette chromosome *mec* (SCC*mec*) and *agr* loci as wells as to establish the pulsotypes and clonal complexes (CCs) for MRSA and methicillin-sensitive *S*. *aureus* (MSSA) outbreaks associated with the operating room (OR) at a pediatric hospital. Twenty-five clinical strains of *S. aureus* (19 MRSA and 6 MSSA strains) were recovered from the outbreak (patients, anesthesia equipment, and nasopharyngeal exudates from external service anesthesia technicians). These clinical *S*. *aureus* strains were mainly resistant to benzylpenicillin (100%) and erythromycin (84%) and were susceptible to vancomycin and nitrofurantoin. The SCC*mec* type II was amplified in 84% of the *S*. *aureus* strains, and the most frequent type of the *agr* locus was *agrII*, which was amplified in 72% of the strains; however, the *agrI* and *agrIII* genes were mainly detected in MSSA strains. A pulsed-field gel electrophoresis (PFGE) analysis grouped the 25 strains into 16 pulsotypes (P), the most frequent of which was P1, including 10 MRSA strains related to the anesthesia equipment, external service anesthesia technicians, and hospitalized patients. Multilocus sequence typing (MLST) identified 15 sequence types (STs) distributed in nine CCs. The most prevalent ST was ST1011, belonging to CC5, which was associated with the SCC*mec* type II and *agrII* type. We postulate that the external service anesthesia technicians were MRSA carriers and that these strains were indirectly transmitted from the contaminated anesthesia equipment that was inappropriately disinfected. Finally, the MRSA outbreak was controlled when the anesthesia equipment disinfection was improved and hand hygiene was reinforced.

## Introduction

*Staphylococcus aureus* is a Gram-positive coccus that normally colonizes the skin and mucous membranes of healthy individuals. *S. aureus* is considered an important opportunistic pathogen in humans that is mainly associated with healthcare-associated infections (HCAIs) (Kallen et al., 2010; Lindsay, 2013; Turner et al., 2019). HCAIs from this bacterium range from mild skin and soft tissue infections to life-threatening diseases, such as endocarditis, osteomyelitis, pneumonia, and bacteremia (McGuinness et al., 2017). In pediatric patients, pneumonia has been associated with mechanical ventilation and bacteremia with high morbidity and mortality rates; furthermore, most cases of pneumonia in this population are associated with methicillin-resistant *S. aureus* (MRSA) (Hassoun et al., 2017; McGuinness et al., 2017; De la Rosa-Zamboni et al., 2018). Approximately 30% of bacteremia’s associated with MRSA are manifested as sepsis or septic shock, which are associated with a significantly high mortality rate in the pediatric intensive care unit (Hassoun et al., 2017). The prevalence of MRSA strains in Mexico of 21.4% has been linked to respiratory infections and significant increases in the prevalence of immunocompromised patients and patients with underlying respiratory diseases (Chacon-Cruz et al., 2019; Garza-González et al., 2019). High prevalence rates of infections associated with hospital-acquired MRSA (HA-MRSA) have been reported worldwide since the sixties (Kallen et al., 2010; Stefani et al., 2012; Lindsay, 2013; De la Rosa-Zamboni et al., 2018; Turner et al., 2019).

Resistance to methicillin in MRSA strains has been related to clonal variants of staphylococcal cassette chromosome *mec* (SCC*mec*), which confers resistance to all β-lactam antibiotics, including flucloxacillin, cephalosporins, and carbapenems. SCC*mec* contains the *mecA* gene that encodes a penicillin-binding protein (penicillin-binding protein-PBP2a) and has low affinity for broad-spectrum β-lactam antibiotics (Lindsay, 2013). SCC*mec* is a mobile genetic element of 20–68 kb that contains the *mec* gene complex, *ccr* gene complex, and J region. Due to the high diversity of structural organization and genetic content, 14 types of SCC*mec* have been identified in MRSA strains. SCC*mec* type I, II, III, IVa, and V have been described with more frequency in the last decade (Lakhundi and Zhang, 2018; Urushibara et al., 2020). SCC*mec* typing has facilitated the differentiation of HA-MRSA strains from community-acquired MRSA (CA-MRSA). HA-MRSA strains frequently contain SCC*mec* types I and II; meanwhile, SCC*mec* type IV is most frequently detected in CA-MRSA strains (Deurenberg and Stobberingh, 2008; Hassoun et al., 2017).

Methicillin-resistant *Staphylococcus aureus* strains express several virulence factors through the *agr* (accessory gene regulator) system, a quorum-sensing system that functions as a master regulator of virulence. The *agr* system senses the bacterial population density of MRSA by concentrating a cyclic signaling peptide and translates this information into a specific pattern of gene expression (Jenul and Horswill, 2018). Several virulence factors are regulated by the *agr* system, such as alpha-toxin (Hla), gamma-hemolysin (Hlg), cysteine proteases (ScpA and SspB), serine proteases (SplA-F and SspA), leucocidins (LukAB and LukGH), and lipase (Geh). In contrast, protein A (Spa), cell wall secretory protein (IsaA), and surface receptors (MnhA, MnhF, and MnhG) are deregulated by RNAIII from the *agr* system (Jenul and Horswill, 2018). The typing of polymorphisms in the *agr* system has facilitated the identification and classification of MRSA clinical isolates in Europe, America, India, Vietnam, and Iran (Hetem et al., 2016; Goudarzi et al., 2017; Aggarwal et al., 2019; Nguyen et al., 2020). Although the *agr* system is conserved in most strains of *S. aureus*, four polymorphisms in the *agr* locus have been identified based on the variable region between AgrD, the C-terminal portion of AgrB, and the N-terminal portion of AgrC (Gilot et al., 2002). The types of *agr* have facilitated the classification of clinical and environmental MRSA isolates into four different groups: *agrI*, *agrII*, *agrIII*, and *agrIV* (Jarraud et al., 2000). In the Mexican pediatric population, HA-MRSA strains derived from bacteremia have been mainly associated with the *agrII* and *agrI* types (Cazares-Dominguez et al., 2015). The molecular characterization of hospital MRSA strains employing pulsed-field gel electrophoresis (PFGE) and multilocus sequence typing (MLST) has enabled differentiation of strains from various sources (Hassoun et al., 2017). The clinical strains of MRSA have been described based on their clonal complex (CC) and/or sequence type (ST). The main CCs identified around the world are CC5, CC8, CC22, CC30, CC45, and ST239 (Stefani et al., 2012). However, MRSA strains associated with CC398 have rapidly emerged in recent years as an important cause of infections in humans, chickens, pigs, and other farm animals (Price et al., 2012). CC398 is the most common livestock-associated MRSA (LA-MRSA) strain worldwide that has been observed to colonize pigs and veal calves, and has been identified in poultry and horses (Vanderhaeghen et al., 2010; Van den Eede et al., 2013). Characteristic resistance genes detected in CC398 LA-MRSA include genes encoding resistance to trimethoprim, tetracycline, macrolides, lincosamides, gentamicin, ciprofloxacin, and trimethoprim-sulfamethoxazole, as well as the antibiotics found in animal feed (Argudín et al., 2011). The spectrum of infections with strains of CC398 in humans ranges from minor localized infections such as abscesses and surgical site infections (SSIs) to urinary tract infections (UTIs) and wound infections. The impact of this clone appears to be low at the moment; however, with its ability to procure genetic elements, such as genes encoding virulence factors and antibiotic resistance genes, it may pose a considerable threat to human health in the future (Lakhundi and Zhang, 2018). The aim of this study was to performing molecular typing of the SCC*mec* and *agr* loci and establish pulsotypes (P) and CCs of MRSA and methicillin-sensitive *S*. *aureus* (MSSA) outbreaks associated with the operating room (OR) at a pediatric hospital.

## Materials and Methods

### Clinical Strains of. *S. aureus*

This study included a set of 25 strains (19 MRSA and 6 MSSA) recovered from a hospital outbreak at the Children’s Hospital of Mexico Federico Gómez (HIMFG), Mexico City, Mexico. The *S. aureus* strains collected were recovered from the several sources. (1) Ten clinical strains of MRSA were recovered from the bloodstream of 10 pediatric patients who were treated in the service areas of cardiovascular surgery (CS), general surgery (GS), neurology (NR), internal medicine (IM), surgery recovery (SR), and the surgical therapy (STP) unit during the period from January to October 2017. These patients underwent a surgical procedure in OR5 and OR6, and a venous catheter was placed in each of them. (2) Five environmental strains of MRSA were obtained from the surface sampling of the anesthesia equipment confined to OR5 and OR6. (3) Four clinical strains of MRSA were isolated from nasopharyngeal exudates of external service anesthesia technicians. (4) Six clinical strains of MSSA were isolated from nasopharyngeal exudates of external service anesthesia technicians and two physicians responsible for the anesthesia team in OR5 and OR6 of the HIMFG. All strains were isolated from different patients, different anesthesia technicians, and different locations. Strains isolated from the same patient, anesthesia technician and place were discarded and a single strain was selected. The clinical strains of MRSA and MSSA were identified with the MALDI-TOF VITEK^®^ MS Microbial Identification System (bioMérieux, Marcy-l’Étoile, France).

### Bacterial Growth

The MRSA and MSSA clinical strains were preserved in skim milk medium (Difco^TM^ BD, 1 Becton Drive, Franklin Lakes, NJ, United States) and stored in a −70°C freezer until further processing. The strains were recovered in blood agar (Dibico^®^, CDMX, Mexico) supplemented with 5% sheep blood (Difco^TM^ BD, 1 Becton Drive, Franklin Lakes, NJ, United States) for 24 h at 37°C.

### Antibiotic Susceptibility

Antibiotic susceptibility was determined by calculating the minimum inhibitory concentration (MIC) using the automated VITEK^®^ 2 method (bioMérieux). The groups of antibiotics included in this study were suggested by CLSI-2020 (Clinical Laboratory Standards Institute, 2020) as follows: penicillinase-labile penicillins (BZ, benzylpenicillin), penicillinase-stable penicillins (OXA, oxacillin), glycopeptides (VA, vancomycin), aminoglycosides (GM, gentamicin), macrolides (E, erythromycin), tetracyclines (TE, tetracycline), fluoroquinolones (CIP, ciprofloxacin; LEV, levofloxacin; MFX, moxifloxacin), nitrofurantoins (F/N, nitrofurantoin), lincosamides (CLI, clindamycin), folate pathway antagonists (TSX, trimethoprim-sulfamethoxazole), ansamycins (RM, rifampin), streptogramins (QD, quinupristin-dalfopristin), oxazolidinones (LZD, linezolid), and glycylcyclines (TIG, tigecycline). The *S. aureus* ATCC 29213 strain was used as a control. The MIC results were compared with the CLSI-2020 tables, and the multidrug resistance (MDR) profiles of the clinical strains were determined.

### Detection of β-Lactamase Production and Methicillin Resistance

Oxacillin resistance was evaluated by determining the MIC using the automated VITEK^®^ 2 method (bioMérieux) according to the manufacturer’s instructions. Briefly, strains with values of ≥4 μg/mL are considered resistant to methicillin and strains with MICs ≤2 μg/mL are considered susceptible, according to CLSI-2020. The production of β-lactamases was determined using the chromogenic cephalosporin test or Cefinase^TM^ test of Becton-Dickinson (Franklin Lakes, NJ, United States) according to the manufacturer’s instructions.

### Assessment of *mecA*-Mediated Oxacillin Resistance Using the Cefoxitin Disk Diffusion Assay

*S. aureus* strains were grown on 5% sheep blood agar and incubated for 18 to 24 h. A bacterial suspension in a physiological saline solution was adjusted to a turbidity of 0.5 using a MacFarland nephelometer (1.5 × 10^8^ bacteria/mL). The bacterial suspension was seeded on Mueller-Hinton agar (MHA) plates. The bacterial inoculum was absorbed after 3 to 5 min, and 30 μg cefoxitin discs were placed in the center of each plate and incubated at 37°C for 18 h. The clinical strains of *S. aureus* positive for *mecA* produced inhibition halos ≤21 mm and strains negative for *mecA* produced inhibition halos ≥22 mm. Cefoxitin resistance was confirmed by testing with the VITEK^®^ 2 platform (bioMérieux). MIC values of 4 μg/mL were considered positive for *mecA*, and values ≤4 μg/mL were negative for *mecA*, according to the CLSI-2020.

### Typing of *agr* and SCC*mec*

The characterization of the *mecA* gene, *agr* locus (*agrI*, *agrII*, *agrIII*, and *agrIV*), and SCC*mec* types (I, II, III, and IVa) was performed using multiplex PCR (Gilot et al., 2002; Zhang et al., 2005; Cazares-Dominguez et al., 2015). Multiplex PCR assays were carried out with a DNA polymerase for highest accuracy (Pfu-X-Polymerase^TM^, Jena Biosciences, Jena, Germany). The primers and amplification conditions are listed in Supplementary Table S1. Multiple PCR products of SCC*mec* types were purified using the DNA clean and concentrator TM-5 purification kit (Zymo Research, Irvine, CA, United States) and subsequently sequenced by capillary electrophoresis following the Sanger method. The sequences obtained were compared with the sequences NCTC10442 (AB033763.2), N315 (NC_002745.2), and 85/2082 (AB038513.1) of the SCC*mec*I, SCC*mec*II and SCC*mec*III types from *S*. *aureus* strains. The sequences from MRSA strains were analyzed by multiple alignment using the MultAlin interface page^1^ to determine the identity between the strains.

### Diversity Analysis Using PFGE

An analysis of the diversity of MRSA strains was performed using the PFGE methodology, as previously described by Cazares-Dominguez et al. (2015). The agarose blocks containing the chromosomal DNA were digested with 20 U of the SmaI enzyme and subjected to 1% agarose gel electrophoresis. The electrophoretic shift was performed using a CHEF-MAPPER (Bio-Rad, 1000 Alfred Nobel Drive, Hercules, CA, United States) at 200 V (6 V/cm) for 22 h at 14°C, with an initial pulse of 5 s and an end pulse of 35 s. The gels containing the DNA fragments were stained with a 1.0 mg/mL ethidium bromide solution and visualized using a gel imaging system (ChemiDoc MP System, Bio-Rad^®^). The DNA fragment patterns generated by PFGE were analyzed using the NTSYS program version 2.0 (Applied Biostatistics, Setauket, New York, NY, United States) with the Sørensen-Dice coefficient de similitude and the unweighted pair group method with an arithmetic mean (UPGMA) clustering system. A dendrogram analysis allowed us to identify the clonality and development of *S. aureus* associated with hospital outbreaks (Tenover et al., 1995).

### MLST Assays

Genomic DNA was extracted with the Quick-DNA fungal/bacterial Midiprep kit (Zymo Research, Irvine, CA, United States) according to the manufacturer’s instructions. Seven housekeeping genes were amplified by endpoint PCR using the Pfu-X enzyme from Jena Biosciences (Jena, Germany). The genomic DNA (100 ng) of each strain was used to amplify the seven housekeeping genes with MLST^2^ according to the Oxford scheme, which included a*rcC* (carbamate kinase), *aroE* (shikimate dehydrogenase), *glpF* (glycerol kinase), *gmk* (guanylate kinase), *pta* (phosphate acetyltransferase), *tpi* (triosephosphate isomerase), and *yqi* (acetyl coenzyme A acetyltransferase). Occasionally, some difficulties in assigning allele numbers for the *gmk* locus have been reported (Larsen et al., 2012). Therefore, every primer was analyzed, and when differences in alignments were observed, the primer was modified in this study. The primers used to amplify these housekeeping genes are listed in Supplementary Table S1. The amplification products were purified with the DNA clean and concentrator-100 kit (Zymo Research^®^) and sequenced with the Sanger sequencing method using the BigDyeTerminator v3.1 Cycle Sequencing kit (Applied Biosystems, Foster City, CA, United States) and the capillary sequencer ABI 3500 Genetic Analyzer (Applied Biosystems^®^). The sequences obtained from each gene were analyzed with chromatograms using Chromas V2.6.6 and Bioedit v7.0.5.3 software. The edited sequences were compared with the *S. aureus* MLST databases (see text footnote 2) to determine the STs. The CCs associated with MRSA and MSSA were analyzed using PHYLOViZ software, which is based on the eBURST algorithm. The data generated were compared with the CCs of MRSA reported in the Bacterial Isolate Genomes Sequence database (BIGSdb^3^) to determine its global distribution (Jolley and Maiden, 2010).

### Disinfection With Ultraviolet-C (UV-C) Light

Ultraviolet-C disinfection was performed using equipment designed with four UV-C lamps (wavelength of 240 nm) at 1.67 m and provides 360 degrees of UV-C radiation. The four UV-C lamps provide an infrared motion sensor at the base of every lamp that turns off when movements are detected. In addition, only 3 min are needed to reduce the log of MRSA counts by >5.69 and 5 min for other bacteria in a space with a diameter of 4.8 m. The equipment was operated in five to seven positions, according to the OR size.

## Results

### Outbreak Description

**FIGURE 1:**
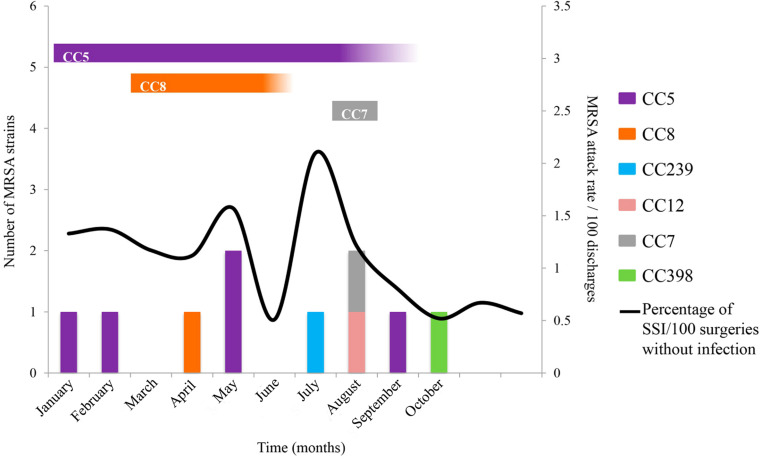
Outbreaks associated with MRSA strains from environmental, nostril, and clinical sources. UV-C was implemented at the end of May twice a week after the two cases occurred in the OR, and patients who developed SSIs were treated. In August, the ORs were sanitized daily with UV-C. MRSA strains were related to several CCs and areas of isolates, CC5 (isolated from ANT, ANM, LT, ECP, and two anesthesia technicians), CC789 (isolated from nostril of a physician and the belongings), and CC8 (isolated from nostril of an anesthesia technician). CC789 and CC7 are closely related and are presented in turquoise blue; likewise, CC8 is related to CC239 and presented in orange. The vertical bars indicate the infected patients, and the black line indicates the SSI rate. The horizontal bars indicate the CC isolates from environmental or nostril samples.

Hospital of Mexico Federico Gómez is a pediatric teaching hospital with a hand hygiene program. From January to October 2017, 10 patients were infected with MRSA strains, and a rate of 0.15/100 cases was observed compared to a rate of 0.02/100 discharges during 2016. The first case of MRSA (839BS strains) occurred as SSI and central line-associated bloodstream infection (CLABSI) after surgery performed in an OR. The other four subsequent patients [one patient with pneumonia (483BS strain), two patients with CLABSI (671BS and 81BS strains) and postsurgical mediastinitis (3BS strain)] were hospitalized and treated by different services (Table 1). The only characteristic shared by these patients was that they underwent surgery or placement of a catheter in OR5 or OR6. In the last 4 months, hand hygiene adherence decreased from 85% to 50% and was accompanied by an increase in the SSI rate from a baseline of 0.5 to 2.3/100 surgeries (Figure 1). Therefore, hand hygiene and SSI prevention measures were reinforced using antibiotic prophylaxis and antisepsis.

**TABLE 1:** Complete results for the 25 strains of *S. aureus* included in the study.

Data isolation					Gene typing				MLST		PFGE	Antibiotic resistance										
Strains**	Ward	IS	S	Date	MRSA	*mecA*	SCC*mec* type	*agr* type	ST	CC	Pulsotype	BZ	FOX	OXA	GM	E	TE	CIP	LEV	MFX	CLI	CLI-Ind
**Pediatric patients**																						
3BS	CS	M/SB	BS	05/15/2017	+	+	II	II	1011	5	P1	R	R	R	S	R	S	R	R	R	R	S
81BS	GS	CRB	BS	05/18/2017	+	+	II	II	143	5	P4	R	R	R	S	R	S	R	R	R	R	S
483BS	NR	PN/SB	BS	02/16/2017	+	+	I, II, III	II	744	5	P7	R	R	R	S	R	S	R	R	R	R	S
671BS	IM	CRB	BS	04/03/2017	+	+	II	II	345	8	P6	R	R	R	S	R	S	R	R	R	R	S
839BS	SR	SSI/SB	BS	01/25/2017	+	+	II	II	1011	5	P1	R	R	R	S	R	S	R	R	R	R	S
585BS	NR	VEN	BS	09/13/2017	+	+	II	II	1011	5	P2	R	R	R	S	R	S	R	R	R	R	S
924BS*	IM	PN	BS	08/22/2017	+	+	II	NT	692	12	P3	R	R	R	S	R	S	R	R	R	R	S
301BS	IM	CRB	BS	10/10/2017	+	+	II	II	1966	398	P5	R	R	R	S	R	S	R	R	R	R	S
567BS	STP	CRB	BS	08/09/2017	+	+	I, II, III	II	5538	7	P8	R	R	R	R	R	S	R	R	R	R	S
836BS*	CS	M	BS	07/17/2017	+	+	II, III	NT	161	239	P9	R	R	R	S	R	S	R	R	R	R	S
**Anesthesia technical staff**																						
4AT	AT	HW	NPS	06/12/2017	+	(−)	(−)	II	5080	5	P10	R	R	R	S	R	S	R	R	R	R	S
6AT	AT	HW	NPS	06/12/2017	(−)	(−)	NT	II	4695	5	P11	R	S	S	S	S	R	S	S	S	S	S
8AT	AT	HW	NPS	06/12/2017	(−)	+	II	III	729	8	P14	R	S	S	S	R	S	S	S	S	S	S
9AT	AT	HW	NPS	06/12/2017	(−)	+	II	I	544	1434	P12	R	S	S	S	S	S	S	S	S	S	S
10AT	AT	HW	NPS	06/12/2017	+	+	II	II	1011	5	P1	R	R	R	S	R	S	R	R	R	R	S
15AT	AT	HW	NPS	06/12/2017	(−)	(−)	NT	I	1163	30	P15	R	S	S	S	R	S	S	S	S	R	R
16AT	AT	HW	NPS	06/12/2017	+	+	II	II	1011	5	P1	R	R	R	S	R	S	R	R	R	R	S
18AT	AT	HW	NPS	06/14/2017	+	+	II	II	1011	5	P1	R	R	R	S	R	S	R	R	R	R	S
25PH	PH	HW	NPS	06/14/2017	(−)	(−)	NT	I	1159	789	P13	R	S	S	S	S	S	S	S	S	S	S
26PH	PH	HW	NPS	06/14/2017	(−)	+	II	III	3667	30	P16	R	S	S	S	S	S	S	S	S	S	S
**Environments**																						
OR5-ANT	EV	NA	ANT	06/06/2017	+	+	II	II	1011	5	P1	R	R	R	S	R	S	R	R	R	R	S
OR5-ANM	EV	NA	ANM	06/06/2017	+	+	II	II	1011	5	P1	R	R	R	S	R	S	R	R	R	R	S
OR5-LT	EV	NA	LT	06/06/2017	+	+	II	II	1011	5	P1	R	R	R	S	R	S	R	R	R	R	S
OR6-ANM	EV	NA	ANM	06/06/2017	+	+	II	II	1011	5	P1	R	R	R	S	R	S	R	R	R	R	S
OR6-ECP	EV	NA	ECP	06/06/2017	+	+	II	II	1011	5	P1	R	R	R	S	R	S	R	R	R	R	S

The general characteristics of the strains, isolate source, gene typing, characterization using PFGE and MLST, and antimicrobial resistance are shown. *Deceased patient. **All strains were isolated from different patients, different anesthetists, and different locations. W, ward; IS, infection site; S, sample; BS, bloodstream; MRSA, methicillin-resistant *S. aureus*; *mecA*, gene that encodes a penicillin-binding protein (PBP2a); SCC*mec*, staphylococcal cassette chromosome *mec*; *agr*, accessory gene regulator system; ST, sequence type; CC, clonal complex; BZ, benzylpenicillin; FOX, cefoxitin; OXA, oxacillin; GM, gentamicin; E, erythromycin; TE, tetracycline; CIP, ciprofloxacin; LEV, levofloxacin; MFX, moxifloxacin; CLI, clindamycin; CLI-ind, inducible-clindamycin; CS, cardiovascular surgery; GS, general surgery; NR, neurology; IM, internal medicine; SR, surgical recovery; STP, surgical therapy; AT, anesthesia technician; PH, physician; EV, environment; NPS, nasopharyngeal swab; OR5, operating room No. 5; OR6, operating room No. 6; ANT, anesthesia table; ANM, anesthesia monitor; LT, laparoscopy tower; ECP, extracorporeal circulation pump; M/SB, mediastinitis/secondary bacteremia; CRB, catheter-related bacteremia; PN/SB, pneumonia/secondary bacteremia; SSI/SB, surgical site infection/secondary bacteremia; VEN, ventriculitis; PN, pneumonia; M, mediastinitis; HW, healthy worker; (+), positive; (−) negative; NT, not typeable; NA, not applicable.

Nasopharyngeal exudates were collected from the medical staff of HIMFG who were negative for MRSA. Subsequently, OR cultures (walls, surgical table, and doors) were generated, which were also negative. Finally, the laparoscopy tower equipment (LT), extracorporeal circulation pump (ECP) and anesthesiology machine were cultured, and were positive for MRSA. Subsequently, nasopharyngeal cultures were collected from external service anesthesia technicians who exclusively maintain the anesthesiology machines; 50% (4/8) were positive for MRSA strains. Disinfection was performed *in situ* with a prefabricated superoxidase solution, according to the CDC guidelines (Guideline of Disinfection and Sterilization in Healthcare Facilities, 2008). The cleanliness and disinfection of the OR improved after the cleaning of the laparoscopy tower and extracorporeal circulating pump; however, contamination by MRSA strains in the anesthesia machine did not change.

Another five cases of MRSA occurred: one patient with SSI and mediastinitis (836BS^∗^ strain), two patients with CLABSI (567BS and 301BS strains), one patient with postsurgical pneumonia (924BS^∗^ strain) and one patient with effusion (585BS strain). Unfortunately, two patients died from the MRSA infection (Table 1). The MRSA outbreak coincided with an increase in SSIs from 0.8% to 1.6% in the same period. Based on this observation, UV-C disinfection was started regularly in all ORs from Monday to Friday, with a coverage of approximately 70%.

### *S. aureus* Strains Identified During the Outbreak Were MRSA

**FIGURE 2:**
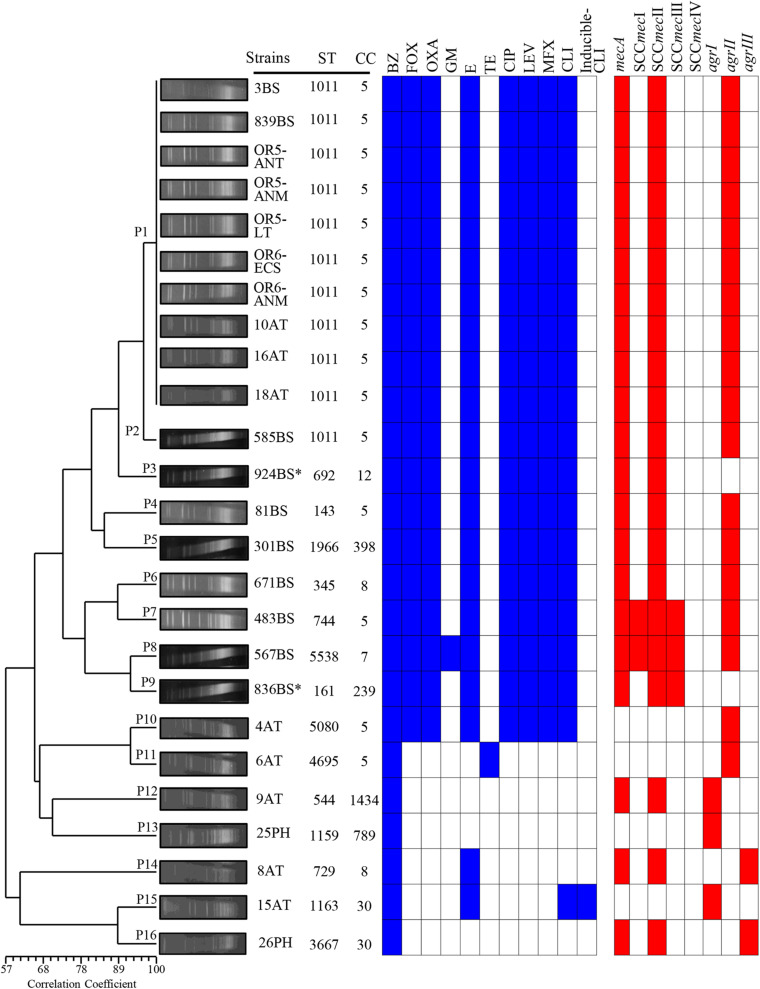
Dendrogram of the PFGE pulsotypes in 25 clinical strains of *S. aureus* associated with an outbreak due to contamination of the anesthesia equipment. The diversity analysis was performed using NTSYS-pc software (version 2.0, Applied Biostatistics, Inc., NY, United States). The absence/presence matrix was evaluated based on the Sørensen-Dice coefficient using the UPGMA algorithm as a grouping method. The dendrogram was evaluated using the cophenetic correlation coefficient obtained with the Mantel test, which indicated the dispersion of the data and showed a value of *r* = 0.87358. The pulsotypes (P), antibiotic resistance, SCC*mec* types and *agr* locus are indicated in a graph. Blue squares indicate resistance, and red squares indicate the presence of the gene. BS, bloodstream; AT, anesthesia technician; PH, physician; OR5, operating room 5; OR6, operating room 6; ANT, anesthesia table; ANM, anesthesia monitor; LT, laparoscopy tower; ECP, extracorporeal circulation pump.

The clinically isolated *S*. *aureus* strains were tested for antimicrobial susceptibility, and the following results were obtained: 100% (25/25) were resistant to penicillinase-labile-penicillins (BZ), 84% (21/25) were resistant to macrolides (E), 80% (20/25) were resistant to lincosamides (CLI), 76% (19/25) were resistant to penicillinase-stable penicillin (OXA), 76% (19/25) were resistant to fluoroquinolones (CIP, LEV, and MFX), 4% (1/25) were resistant to aminoglycosides (GM), and 4% (1/25) were resistant to tetracyclines (TE) and inducible (CLI) (Table 1). All strains were susceptible to VA and F/N. Nineteen MRSA strains were isolated from 10 patients with bloodstream infections, four were isolated from nasopharyngeal swabs of anesthesia technicians, and five were isolated from equipment that included the anesthesia table (ANT), anesthesia monitor (ANM), laparoscopy tower (LT), and extracorporeal circulation pump (ECP) in OR5 and OR6. The MRSA strains showed resistance to at least three families of antibiotics (Figure 2 and Table 1). In addition, six MSSA strains were obtained from nasopharyngeal swabs of four external anesthesia technicians and two physicians associated with the surgery service (Figure 2 and Table 1).

### SCC*mec* Type II and *agrII* Gene Were the Most Common Detected in *S. aureus* Strains

Eighty-five percent (18/21) of *mecA*-positive *S*. *aureus* strains only harbor SCC*mec* type II. Furthermore, 9.52% (2/21) of *mecA*-positive *S*. *aureus* strains amplified SCC*mec* type I and 14.28% (3/21) SCC*mec* type III (Figure 2 and Table 1). The *agrII* gene was the most frequent type identified in 76% (16/21) of *S*. *aureus* strains, compared to *agrIII* at 9.5% (2/21) and *agrI* at 4.8% (1/21). The *agrI* gene was mainly detected in MSSA, in contrast to the *agrII* gene, which was most predominant in MRSA strains. The SCC*mec* type IV and *agrIV* were not detected in the *S*. *aureus* strains tested in this study. Interestingly, the three types of SCC*mec* (I, II, and III) amplified in the 483BS and 567BS MRSA strains; while two types of SCC*mec* (II and III) amplified only in the 836BS^∗^ strain (Figure 2 and Table 1). Sequencing analysis confirmed also the presence of two SCC*mec* types (II and III) in strain 836BS^∗^, and three types of SCC*mec* (I, II, III) in strains 483BS and 567BS MRSA, which showed among 94 to 100% identity when compared with the reference strains.

### Genetic Diversity of Clinical and Environmental *S. aureus* Strains

The macrorestriction pattern obtained using PFGE showed fragments ranging from 48.5 to 533.5 kb. The 25 strains were grouped into 16 pulsotypes with a cophenetic correlation coefficient of 0.87358. P1 harbored 10 MRSA strains with identical macrorestriction patterns that were isolated from patients with bloodstream infections (3BS and 839BS), equipment in OR5 and OR6 strains (OR5-ANT, OR5-ANM, OR5-LT, OR6-ECP, and OR6-ANM), and external service anesthesia technicians (10AT, 16AT, and 18AT) (Figure 2 and Table 1). Based on this finding, the external service anesthesia technicians were carriers of the MRSA strains and transmission was due to indirect contamination of the anesthesia equipment by anesthesia technician and the patients subsequently acquired the bacteria.

Other MRSA strains were grouped with ≥80% correlation coefficients in P2 (585BS), P3 (924BS^∗^), P4 (81BS), and P5 (301BS). Moreover, MRSA strains were assigned to P6 (671BS), P7 (483BS), P8 (567BS), P9 (836BS^∗^), and P10 (4AT) with a correlation coefficient of less than 80%. The other pulsotypes contained MSSA strains. Additionally, P1 included the first MRSA strain, which was isolated on 01/25/2017, and P5 included the last MRSA strain, which was isolated on 10/10/2017. Interestingly, the MRSA strain (585BS) isolated on 09/13/2017 was closely related to strains grouped in P1, suggesting a common ancestor (Figure 2).

### CC5 Contained the Greatest Number of MRSA Strains

**FIGURE 3:**
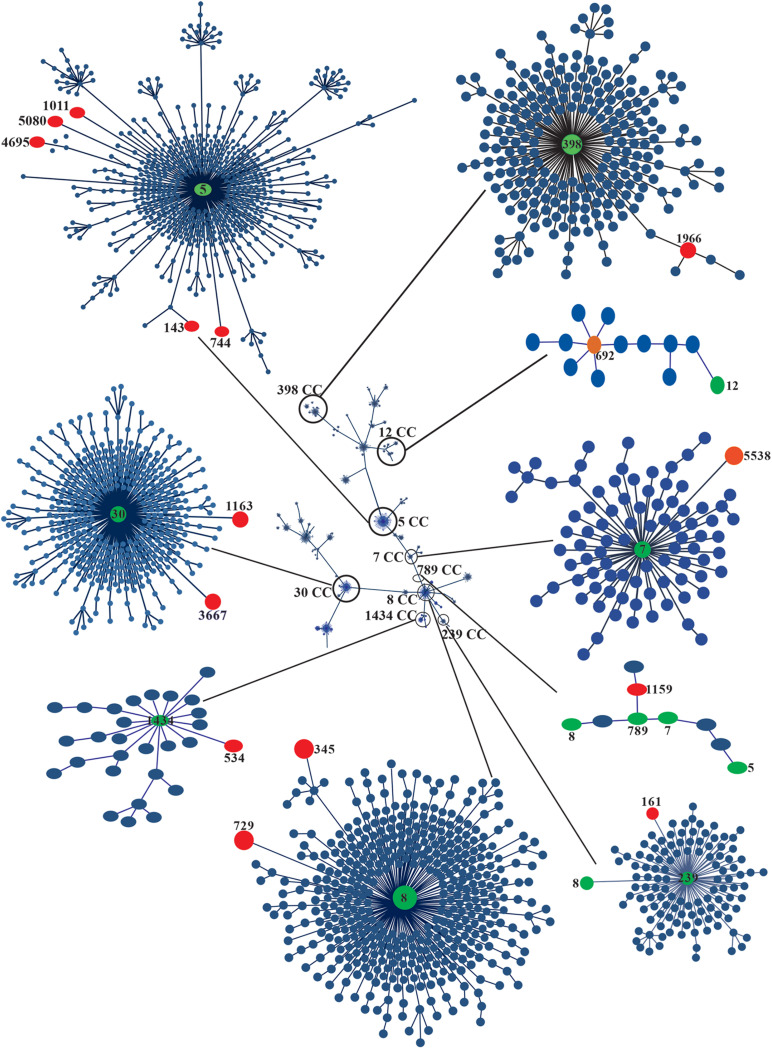
eBURST analysis of 25 clinical strains of MRSA. The phylogenetic relationships of the CCs and STs associated with the outbreak are shown in circles of different colors. The global population distribution of the strains is also shown. Minimum spanning trees of the clinical strains of MRSA and MSSA were built using PHYLOViZ Online with the goeBURST algorithm based on the sequences reported in the MLST database. The STs of the strains are indicated with red circles, and the CCs are indicated with green circles.

Multilocus sequence typing was performed to obtain the ST of *S*. *aureus* strains. Fifteen STs were identified and distributed in nine CCs according to MLST (Figure 2 and Table 1). ST1011 was the most prevalent at 44% (11/25), followed by ST692, ST143, ST1966, ST345, ST744, ST5538, ST161, ST5080, ST4695, ST544, ST1159, ST729, ST1163, and ST3667 identified in the remaining strains (Supplementary Table S2). SCC*mec* type II and *agrII* genes were amplified in MRSA ST1011 (CC5), ST143 (CC5), ST1966 (CC398), and ST345 (CC398). Interestingly, ST744 (CC5) and ST5538 (CC7) harbored MRSA strains with three SCC*mec* types and *agrII* genes compared with ST161 (CC239) MRSA strains with two SCC*mec* types (II and III) and *agrII* genes. CC5 harbored most of the MRSA strains [67% (14/21)] and was detected with the SCC*mec* type II and *agrII* genes. According to the results obtained using PFGE, the strains grouped in P1 and P2 showed the same ST, suggesting a clonal relationship (Figures 2, 3 and Table 1).

## Discussion

The relevance of MRSA in the hospital has been related to infections associated with health care, such as pneumonia, bacteremia, and wound infections (Rago et al., 2012; De la Rosa-Zamboni et al., 2018; Friedrich, 2019; Gibson, 2019). The cross-contamination by hospital equipment confined to specific surgical areas with MRSA strains residing in the nasopharynx by health staff has not been extensively studied in hospitals in Mexico. Our study enabled us to suggest the origin of the outbreak and its relationship with the anesthesia technician. The similarity observed between strains harboring two P (P1 and P2) and ST1011 suggest that the outbreak originated with the contamination of anesthesia equipment by an MRSA strain originating in the nasopharynx of the anesthesia technical staff. Perioperative nasal transport of MRSA is common in ORs, which has been directly related to cases of postoperative bacteremia in patients undergoing surgery, due to the reduced incidence of SSIs (Loftus et al., 2018).

In our study, the control of MRSA infections was achieved by UV-C disinfection of all ORs, where the cleaning and sanitizing of anesthesiology equipment were reinforced according to the CDC guidelines (Guideline of Disinfection and Sterilization in Healthcare Facilities, 2008). The patients who entered the OR that had been decontaminated with UV-C (ultraviolet light) did not develop a MRSA infection. Disinfection of contact surfaces, ORs, and isolation rooms using UV-C sources has been a successful tool in reducing and controlling MDR bacterial populations in various hospitals worldwide (Jinadatha et al., 2014; Hosein et al., 2016; Simmons et al., 2018; Villacís et al., 2019).

The MRSA strains isolated in our study (76%) were resistant to at least three groups of antibiotics and susceptible to three antibiotics (VA, TSX, and LZD). This result indicates a constant resistance over time, which may be related to the low horizontal transfer of genes associated with resistance to these antibiotics (Cazares-Dominguez et al., 2015). However, the resistance to other antibiotics (GM and TE) has varied in different regions of the world (Khosravi et al., 2017; Coombs et al., 2018; Seyedi-Marghaki et al., 2019). The resistance of MRSA strains may be associated with the implementation of strategies related to the administration of antibiotics, improvements in infection control procedures (hand hygiene and isolation of patients) and decolonization protocols for patients and health personnel. Strains classified as HA-MRSA frequently contain SCC*mec* types I, II and III; therefore, the *S. aureus* strains of the outbreak are mainly of hospital origin. MRSA-SCC*mec* type II strains have been isolated from healthy children, suggesting the spread of hospital strains to the community setting (Hisata et al., 2005).

Three strains (6AT, 25PH, and 15AT) identified as MSSA did not contain the *mecA* gene and were isolated from the nasopharyngeal cultures of healthy anesthesia technicians and medical personnel, consistent with previous reports (Sarkar et al., 2016; Senok et al., 2020). However, one strain was resistant to OXA and FOX, but negative for the *mecA* gene. Methicillin resistance is associated with the presence of the homologous gene *mecA*_LGA__251_ (*mecC*) and an assay designed to detect the *mecC* gene should be implemented (Stefani et al., 2012).

We isolated three MRSA strains (9AT, 8AT, and 26PH) with oxacillin-sensitive (OS) profiles, although they were *mecA*-positive (OS-MRSA). According to some studies, 50% of clinical MSSA cases represent infections with *mecA*-positive strains with OXA MICs of 1–2 μg/mL (Chen et al., 2012; Conceição et al., 2015). OS-MRSA strains were recovered from healthy anesthesia technicians and medical personnel and expressed SCC*mec* type II. Based on this finding, healthy individuals may be reservoirs of OS-MRSA strains and contribute to transmission to pediatric patients.

Additionally, more than one type of SCC*mec* were amplified from three MRSA strains. Strains 483BS and 567BS amplified three types of SCC*mec* types (I, II, and III), while the 836BS^∗^ strain presented two SCC*mec* types (II and III). Composite strains with different SCC*mec* elements from MRSA and *Staphylococcus pseudintermedius* were described in Japan (Descloux et al., 2008; Ishihara et al., 2010; Youn et al., 2011). Clinical MRSA strains carrying two to three different types of SCC*mec* have been previously reported (Nagasundaram and Sistla, 2019). SCC*mec* types I through V are frequently identified by multiplex PCR, which has been considered a good tool (Nagasundaram and Sistla, 2019). In this study, were amplified three types of SCC*mec* (I, II, and III) in three MRSA strains; however, due to the high variability of SCC*mec* types a sequencing analysis of the amplified products confirmed the presence of the different types of SCC*mec* (Lakhundi and Zhang, 2018; Nagasundaram and Sistla, 2019). The presence of multiple types of SCC*mec* is supported by a “multi clone theory,” which suggest that the acquisition of several types of SCC*mec* can occur on multiple occasions, with the integration of additional types of SCC*mec* within the existing ones (Nagasundaram and Sistla, 2019). MRSA strains with different SCC*mec* types may reveal an important mechanism of transmission to humans.

On the one hand, 72% of the MRSA strains were of the *agrII* type, while, the MSSA strains obtained from the nasopharynx of the anesthesia technicians and medical personnel were type *agrI* (12%) and *agrIII* (8%). Similar results were obtained from another collection of MRSA strains, suggesting a close relationship of hospital-origin MRSA strains with the *agrII* type (Cazares-Dominguez et al., 2015). Variable results of the *agr* types as crucial regulatory component of bacterial virulence factor expression have been published in previous studies (Goudarzi et al., 2017; Aggarwal et al., 2019). In the present study, only two outbreak strains were not typed for the *agr* locus, and several studies indicate that the distribution of the *agr* types depends on the geographic region (Goudarzi et al., 2017; Aggarwal et al., 2019). Specific *agr* types are associated with specific clinical features (Ji et al., 1997; Sakoulas et al., 2002).

The data obtained using different molecular typing techniques provided relevant information on the diversity and distribution of the MRSA and MSSA strains related to the outbreak. The results of the 16 pulsotypes showed that all MRSA strains from OR5 and OR6, two bloodstream strains (3BS and 839BS), and three nasopharyngeal strains isolated from anesthesia technicians (10AT, 16AT, and 18AT) were P1. Furthermore, P1 showed 90% genetic similarity to P2 (585BS) and P3 (924BS^∗^), indicating that they were closely related and probably started the outbreak. Interestingly, the clinical MRSA strains associated with P4 to P9 were grouped into a separate cluster from the MSSA strains, with <60% similarity between them, indicating that they were distantly related (Tenover et al., 1995).

According to the ST analysis, *S. aureus* strains associated with this outbreak were grouped into nine CCs. CC5 contained the most prevalent MRSA strain, and CC30 was characteristic of the MSSA strains. MRSA strains belonging to CC5 have been reported in different parts of the world (Goudarzi et al., 2017; Aggarwal et al., 2019; Chen et al., 2020; Neradova et al., 2020). CC5-related MRSA has been associated with SCC*mec* type II and a hospital origin, and with the epidemic clone New York/Japan widely distributed worldwide, including in Mexico (Ishihara et al., 2010; Rodríguez-Noriega et al., 2010). The MRSA ST1011 strain has been associated with SCC*mec* I and II in Latin America (Arias et al., 2017). This ST was recently identified in MRSA strains isolated from patients in a tertiary care hospital in Mexico (Negrete-González et al., 2020). Sequence type ST1011 differed from ST5 in the replacement of a nucleotide in the *arcC* gene. Thus, ST1011-II is not a New York/Japan clone, but it may be a variant of this strain that originated in the late 1990s, the period when the CC30 was replaced in Mexico. Based on the available data, ST1011-II-t9364 may be a Mexican variant of the New York/Japan clone, which has increased in prevalence in the last 11 years; however, more studies are required to determine the differences compared with ST5-IIt895 (Arias et al., 2017; Challagundla et al., 2018; Negrete-González et al., 2020). ST239-associated HA-MRSA strains (CC239) display resistance to E, CIP, GM, TE, and CLI (Coombs et al., 2018; Dai et al., 2019). In our study, the death of one of the two patients was related to strains 836BS^∗^, ST161, and CC239. The clones simultaneously presenting SCC*mec* types II and III were resistant to E, CIP, LEV, MFX, and CLI. A prevalence of 6–13% of MRSA strains has been associated with ST30 CC30, which were classified as a community clone with characteristics of resistance only to β-lactam antibiotics and sporadic resistance to E, CIP, GM, RM, and TE (Coombs et al., 2018). In our study, 8% (15AT and 26PH strains) of the strains were associated with STs 1163 and 3667, both of which included in CC30. The MSSA strains were resistant only to β-lactams, and the 15AT strain was resistant to E and CLI, as previously described by Coombs et al. (2018). CC30 and CC5 are clones that are highly prevalent in Mexico (Rodríguez-Noriega et al., 2010). ST398 (CC398) is the most prevalent clone of LA-MRSA and has emerged since 2012 as a major cause of hospital infections in humans (Chen et al., 2012; Price et al., 2012). The CC389-associated strain 301B is resistant to β-lactams and E (Chen et al., 2012), and interestingly, these strains were characterized by the presence of the locus *agrI* and SCC*mec* type II. CC8 is a CA-MRSA clone with a high prevalence in Latin America (Rodríguez-Noriega et al., 2010; Jiménez et al., 2012). In our study, the 671BS and 8AT strains were related to CC8, SCC*mec* II, and *agrII*. Thus, the Mexican CC8 clone may acquire characteristics of a hospital strain.

Methicillin-resistant *Staphylococcus aureus* strains have been identified using various typing methods, such as PFGE, MLST, SCC*mec*, and *spa* typing. The information generated is potentially useful for tracing outbreaks, identifying the source of colonization, and distinguishing between community and hospital strains. A combination of methods may be needed to identify some strains. PFGE has been proposed as the gold standard for MRSA typing due to its discriminatory power and reproducibility, as well as the ease of execution, data interpretation, and availability (Struelens et al., 1992; Bens et al., 2006). The major limitations of this technique are the time-consuming and labor-intensive protocols, the cost of the reagents, and the specialized equipment needed. Additionally, the technique lacks sufficient resolving power to discriminate bands differing in size by 5%, and it generates a limited number of gel bands, making the results difficult to interpret (Weller, 2000; Goering, 2010). Despite these limitations, PFGE remains a useful technique for the characterization of outbreaks and has been extensively used to obtain a better understanding of the epidemiology of both endemic and epidemic MRSA strains (Weller, 2000). In these situations, the data analysis criteria developed by Tenover et al. (1995) have been useful.

Multilocus sequence typing is a molecular tool for creating an evolutionary framework of *S. aureus* strains, and the housekeeping genes analyzed using this technique do not have any direct relationship to the virulence of the strains (Enright et al., 2000; Robinson and Enright, 2003, 2004; Lamers et al., 2011). The major limitations of MLST are the cost and the equipment required for execution. Therefore, it is unlikely to be used as a technique for studying putative outbreaks in a hospital, and its use is limited to large centers involved in global epidemiology studies (Trindade et al., 2003; Van Leeuwen et al., 2003). High-quality sequencing data on all of the seven alleles must be obtained.

The discriminatory power of *spa* typing lies between PFGE and MLST (Malachowa et al., 2005); however, compared to both of the other methods, it is cost-effective, easy to use, rapid and displays excellent reproducibility and stability. Based on these features, it represents the most useful instrument and method of choice for characterizing *S. aureus* isolates at local, national, and international levels (Hallin et al., 2007; Deurenberg et al., 2009). The benefits obtained from performing whole-genome sequencing (WGS) during outbreak detection is the increased sensitivity and enhanced isolate discrimination, as it permits the entire genomic DNA sequence of isolates to be determined and compared rapidly. The evolving dynamics of clones spread from the community and hospitals is only becoming clear with the implementation of WGS studies. WGS is also able to exclude cross-transmission when isolates are different (Humphreys and Coleman, 2019). However, the technique is costly and requires trained staff to analyze and interpret the results.

In conclusion, the SCC*mec* and *agr* types, together with the molecular differentiation by MLST, facilitated the identification of the outbreak strains in ST1011 and CC5, which are widely distributed in Mexico. Furthermore, the PFGE assay allowed us to identify MRSA strains closely related to the outbreak and showed the genetic diversity of MSSA strains. However, new typing techniques, such as *spa* and/or microarrays, will enrich the results of this study. We suggest that indirect contamination of the anesthesia equipment by MRSA resident in the nasopharynx of healthy health care personnel was the initial source of the outbreak. The outbreak was controlled by treating carriers of MRSA and MSSA and by disinfecting the affected areas and equipment in the ORs with UV-C light. Hand hygiene was reinforced as a strategy to reduce MRSA infections. Hand hygiene should be constantly monitored to reduce outbreaks.

## Data Availability Statement

The original contributions presented in the study are publicly available. This data can be found here: https://github.com/JetsiMancilla/MLST-Staphylococcus-aureus.

## Author Contributions

SO, JX-C, AC-C, and DR-Z designed and conceived the experiments. GE-V, JM-R, VE-K, IF-H, SO, and AC-C performed the experiments. SO, AC-C, JM-R, DR-Z, and JX-C analyzed the data. SO, AC-C, IP-O, JA-G, RH-C, CP-L, DR-Z, and JX-C contributed to the reagents, materials, and analysis tools. All authors contributed to the article and approved the submitted version.

## Conflict of Interest

The authors declare that the research was conducted in the absence of any commercial or financial relationships that could be construed as a potential conflict of interest.

## Footnotes

1 http://multalin.toulouse.inra.fr/multalin/

2 https://pubmlst.org/saureus/

3 https://pubmlst.org/software/database/bigsdb/
