# Supplementary material for: Control of Methicillin-Resistant Staphylococcus aureus Strains Associated With a Hospital Outbreak Involving Contamination From Anesthesia Equipment Using UV-C
Source: Front Microbiol. 2020 Dec 14;11:600093. doi: 10.3389/fmicb.2020.600093 (PMC7767929; doi:10.3389/fmicb.2020.600093)
Supplement: Supplementary file 1 [file Table_1.DOCX]

Supplementary Table 1. Primers used for PCR amplification of the *agr* polymorphisms and type of SCC*mec* as well as for MLST.

| **Gene** | **Primer** | **Sequence 5’-3’** | **Reference** |
| --- | --- | --- | --- |
| *agr*I | agr I-F | ATGCACATGGTGCACATGC | Gilot et al. (2002) |
|  | agr I-R | GTCACAAGTACTATAAGCTGCGAT |  |
| *agr*II | agr II-F | ATGCACATGGTGCACATGC | Gilot et al. (2002) |
|  | agr II-R | TATTACTAATTGAAAAGTGGCCATAGC |  |
| *agr*III | agr III-F | ATGCACATGGTGCACATGC | Gilot et al. (2002) |
|  | agr III-R | GTAATGTAATAGCTTGTAAAAAGTGGCCATAGC |  |
| *agr*IV | agr IV-F | ATGCACATGGTGCACATGC | Gilot et al. (2002) |
|  | agr IV-R | CGATAATGCCGTAATACCCG |  |
| *SCCmec*I | mec I-F | GCTTTAAAGAGTGTCGTTACAGG | Zhang et al. (2005) |
|  | mec I-R | GTTCTCTCATAGTATGACGTCC |  |
| *SCCmec*II | mec II-F | CGTTGAAGATGATGAAGCG | Zhang et al. (2005) |
|  | mec II-R | CGAAATCAATGGTTAATGGACC |  |
| *SCCmec*III | mec III-F | CCATATTGTGTACGATGCG | Zhang et al. (2005) |
|  | mec III-R | CCTTAGTTGTCGTAACAGATCG |  |
| *SCCmec*IVa | mec IVa-F | GCCTTATTCGAAGAAACCG | Zhang et al. (2005) |
|  | mec IVa-R | CTACTCTTCTGAAAAGCGTCG |  |
| *SCCmec*V | mec V-F | GAACATTGTTACTTAAATGAGCG | Zhang et al. (2005) |
|  | mec V-R | TGAAAGTTGTACCCTTGACACC |  |
| *mecA* | mec 147-F | GTGAAGATATACCAAGTGATT | Zhang et al. (2005) |
|  | mec 147-R | ATGCGCTATAGATTGAAAGGAT |  |
| Carbamate kinase (*arcC*) | arcF | TTG ATT CAC CAG CGC GTA TTG TC | Enright et al. (2000) |
|  | arcR | AGG TAT CTG CTT CAA TCA GCG |  |
| Shikimate dehydrogenase (*aroE*) | aroF | ATC GGA AAT CCT ATT TCA CAT TC | Enright et al. (2000) |
|  | aroR | GGT GTT GTA TTA ATA ACG ATA TC |  |
| Glycerol kinase (*glp*) | glpF | CTA GGA ACT GCA ATC TTA ATC C | Enright et al. (2000) |
|  | glpR | GAA CCA CCT TTA CCA GCT ATT GG | This study |
| Guanylate kinase (*gmk*) | gmkF | GGA TAA TGA AAA AGG ATT GTT AAT CG | This study |
|  | gmkR | TCA TTA ACTA CAA CGT AAT CGT A | Enright et al. (2000) |
| Phosphate acetyltransferase (*pta*) | ptaF | GTA TTA CCT GAA GGA GAG GAC GAG | This study |
|  | ptaR | GAC CCT TTT GTT GAA AAG CTT AA | Enright et al. (2000) |
| Triosephosphate isomerase (*tpi*) | tpiF | TCG TTC ATT CTG AAC GTC GTG AA | Enright et al. (2000) |
|  | tpiR | TTT GCA CCT TCT AAC AAT TGT AC |  |
| Acetyl coenzyme A acetyltransferase (*yqiL*) | yqiF | CAG CAT ACA GGA CAC CTA TTG GC | Enright et al. (2000) |
|  | yqiR | CGT TGA GGA ATC GAT ACT GGA AC |  |

*agr,* accessory gene regulatory locus; *SCCmec*, staphylococcal cassette chromosome *mec*; the *mecA* gene encodes a penicillin-binding protein (PBP2a) for resistance to broad-spectrum penicillins; F, forward, R, reverse.
