# Supplementary material for: Control of Methicillin-Resistant Staphylococcus aureus Strains Associated With a Hospital Outbreak Involving Contamination From Anesthesia Equipment Using UV-C
Source: Front Microbiol. 2020 Dec 14;11:600093. doi: 10.3389/fmicb.2020.600093 (PMC7767929; doi:10.3389/fmicb.2020.600093)
Supplement: Supplementary file 2 [file Table_2.DOCX]

Supplementary Table 2

| Strain | *arcC* | *aroE* | *glpF* | *gmK* | *pta* | *tpi* | *yqi* | ST | CC | Date of isolation |
| --- | --- | --- | --- | --- | --- | --- | --- | --- | --- | --- |
| 3BS | 126 | 4 | 1 | 4 | 12 | 1 | 10 | 1011 | 5 | 05/15/2017 |
| 839BS | 126 | 4 | 1 | 4 | 12 | 1 | 10 | 1011 | 5 | 01/25/2017 |
| OR5-ANT | 126 | 4 | 1 | 4 | 12 | 1 | 10 | 1011 | 5 | 06/06/2017 |
| OR5-ANM | 126 | 4 | 1 | 4 | 12 | 1 | 10 | 1011 | 5 | 06/06/2017 |
| OR5-LT | 126 | 4 | 1 | 4 | 12 | 1 | 10 | 1011 | 5 | 06/06/2017 |
| OR6-ECS | 126 | 4 | 1 | 4 | 12 | 1 | 10 | 1011 | 5 | 06/06/2017 |
| OR6-ANM | 126 | 4 | 1 | 4 | 12 | 1 | 10 | 1011 | 5 | 06/06/2017 |
| 10S | 126 | 4 | 1 | 4 | 12 | 1 | 10 | 1011 | 5 | 06/12/2017 |
| 16S | 126 | 4 | 1 | 4 | 12 | 1 | 10 | 1011 | 5 | 06/12/2017 |
| 18S | 126 | 4 | 1 | 4 | 12 | 1 | 10 | 1011 | 5 | 06/12/2017 |
| 585BS | 126 | 4 | 1 | 4 | 12 | 1 | 10 | 1011 | 5 | 09/13/2017 |
| 924BS | 12 | 89 | 1 | 1 | 4 | 5 | 90 | 692 | 12 | 08/22/2017 |
| 81BS | 1 | 65 | 1 | 4 | 12 | 63 | 10 | 143 | 5 | 05/18/2017 |
| 301BS | 3 | 35 | 264 | 3 | 26 | 20 | 39 | 1966 | 398 | 10/10/2017 |
| 671BS | 3 | 32 | 1 | 1 | 4 | 4 | 63 | 345 | 8 | 04/03/2017 |
| 483BS | 1 | 4 | 1 | 72 | 12 | 1 | 10 | 744 | 5 | 02/16/2017 |
| 567BS | 5 | 824 | 1 | 4 | 4 | 6 | 3 | 5538 | 7 | 08/09/2017 |
| 836BS | 2 | 3 | 1 | 1 | 4 | 93 | 3 | 161 | 239 | 07/17/2017 |
| 4S | 1 | 4 | 1 | 387 | 12 | 1 | 10 | 5080 | 5 | 06/12/2017 |
| 6S | 1 | 4 | 639 | 4 | 12 | 41 | 10 | 4695 | 5 | 06/12/2017 |
| 9S | 1 | 4 | 1 | 8 | 4 | 4 | 76 | 544 | 1434 | 06/12/2017 |
| 25PH | 3 | 4 | 1 | 4 | 4 | 44 | 3 | 1159 | 789 | 06/14/2017 |
| 8S | 3 | 3 | 1 | 1 | 98 | 4 | 3 | 729 | 8 | 06/12/2017 |
| 15S | 2 | 2 | 2 | 2 | 6 | 61 | 2 | 1163 | 30 | 06/12/2017 |
| 26PH | 2 | 2 | 2 | 281 | 6 | 3 | 2 | 3667 | 30 | 06/14/2017 |
